# Theorizing is for everybody: Advancing the process of theorizing in implementation science

**DOI:** 10.3389/frhs.2023.1134931

**Published:** 2023-03-10

**Authors:** Rosemary D. Meza, James C. Moreland, Michael D. Pullmann, Predrag Klasnja, Cara C. Lewis, Bryan J. Weiner

**Affiliations:** ^1^ Kaiser Permanente Washington Health Research Institute, Seattle, WA, United States; ^2^ Independent Researcher, Seattle, WA, United States; ^3^ Department of Psychiatry and Behavioral Sciences, University of Washington, Seattle, WA, United States; ^4^ School of Information, University of Michigan, Ann Arbor, MI, United States; ^5^ Department of Global Health, University of Washington, Seattle, WA, United States; ^6^ Department of Health Systems and Population Health, University of Washington, Seattle, WA, United States

**Keywords:** theorizing, determinant prioritization, mechanisms, implementation science, causality

## Abstract

There has been a call to shift from treating theories as static products to engaging in a process of theorizing that develops, modifies, and advances implementation theory through the accumulation of knowledge. Stimulating theoretical advances is necessary to improve our understanding of the causal processes that influence implementation and to enhance the value of existing theory. We argue that a primary reason that existing theory has lacked iteration and evolution is that the process for theorizing is obscure and daunting. We present recommendations for advancing the process of theorizing in implementation science to draw more people in the process of developing and advancing theory.

## 1. Introduction

Theories and frameworks (i.e., theoretical products) bring clarity to complex systems within which implementation occurs (1) and provide explicit assumptions that can be collectively tested, validated, or refined (2) (see Table 1 for definitions). As such, they support efficiency in generalizing knowledge across contexts (3). Determinant frameworks commonly describe *what* is likely to impact implementation by defining and organizing determinants while implementation theories often provide explanations for *how* change is believed to occur (2). Theoretical products are often used deductively to guide empirical enquiry, yet we fail to inductively modify theory based on findings (1, 4, 5). In doing so, we miss opportunities to advance theory in light of accumulating evidence, leaving implementation science susceptible to stagnation.

**Table 1:** Terminology.

Terminology	Description
Postulate	A proposition or explanation to be investigated.
Concept	A theoretical entity used in a postulate.
Hypothesis	Data statements that form the basis for testing a postulate.
Theory	“An organized, heuristic, coherent, and systematic set of statements related to significant questions that are communicated in a meaningful whole.” (12)
Determinant framework	Articulate determinants that act as barriers and facilitators that influence implementation outcomes.
Grand theories	Broad theories made up of abstract concepts and postulates. Grand theories tend to be general enough to be widely applicable across contexts.
Middle-range theories	Theories with a narrower scope, less abstract, and have a higher degree of contextualization than grand theories. They fall between working hypotheses and all-inclusive grand theories. Their lower level of generalizability can allow for greater accuracy.
Micro-theory	Narrow scope theories that tend to focus on explaining a specific phenomenon within a particular context or population. These narrow scope theories can include a theory of the problem, such as explaining how determinants jointly impede implementation in a particular context or a theory of the solution, such as a program theory that provides an explanation about how a specific policy, intervention, or project is believed to function.
Theorizing	A process that draws on empirical data to develop, validate, modify or expand theoretical explanations.
Multiple working hypotheses	A process of proposing multiple competing hypotheses that can be tested within a single study.

There has been a call to shift from treating theories as static products to engaging in a process of theorizing that *draws on empirical data* to develop, validate, modify or expand theoretical explanations in implementation science (4). Theorizing, as described here, includes the development of new explanations, but also the refinement of existing theoretical explanations. Everyone has the potential to contribute to theorizing, but many do not explicitly do so. This is partly due to two reasons. First, our understanding of what constitutes a theory is too grand. Others have outlined the characteristics of strong theory, such as clarity in relationships between concepts, explanatory power, and generalizability (6). These characteristics are the aspirational endpoint of good theories, not the starting point.

Second, many do not engage in theorizing due to a failure to recognize opportunities for research to contribute to advancing, refining, or (in)validating theory. When findings conflict with theory, authors rarely question the theory's validity, but rather consider explanations such as weaknesses in study design (7). Researchers should be empowered to challenge theory, regardless of its popularity, prestige, or longevity when warranted by evidence.

Increasing explicit engagement in theorizing will require that researchers are equipped with tools to support theorizing. Inspired by writers like bell hooks who sought to communicate feminist thinking in a way that was accessible to everyone (8), we strive to make clear how theorizing is for everybody. To facilitate this, we draw on the building blocks of theory (9, 10) to describe how empirical research can advance the parsimony and comprehensiveness of theory, and elucidate the boundary conditions under which theory is most accurate. We illustrate how these building blocks can be used to develop micro-theories that provide explanations for how implementation determinants influence implementation. Lastly, we discuss how adopting multiple working hypotheses can discourage the calcification and reification of premature theories by arbitrating between multiple tenable explanations for a phenomenon (11).

## 2. Theorizing in implementation science

### 2.1. Sources for theorizing

Theorizing can be inspired by direct observation or vicariously through the synthesis of existing knowledge. Existing theoretical products in implementation science have stemmed from developers' experience, synthesis of empirical evidence, and drawing on or synthesizing existing theories and frameworks (2). Micro, middle-range, and grand theories can have reciprocal influences on one another. For instance, lower-order theories can be inspired by focusing on a narrow element of a grand theory or, conversely, higher order theories can emerge from synthesis of narrower middle-range and micro theories (13). Whether developing a novel micro-theory from limited empirical observations or modifying a middle-range theory through synthesizing numerous studies, such theorizing can have implications for the full ladder of theories.

### 2.2. Building blocks of theory

The building blocks of theory construction have previously been outlined to describe the attributes of a well-formed comprehensive theory (9, 10). We draw on them to demonstrate how research and reasoning that addresses *any one of these questions* can contribute to advancing theory.

*What* refers to concepts and constructs relevant in explaining a phenomenon. Research can inform the sufficiency and parsimony of middle-range theoretical products by answering the questions *what is missing from the explanation of this phenomenon* and *what is not contributing to explaining this phenomenon?* While implementation science must not stop at classifying determinants (14), determinant frameworks are critical in organizing the science. They influence study questions, hypotheses, measurement, and implementation targets (2, 15). Determinant frameworks were informed by varying degrees of evidence (2), so assessing the validity of their postulated determinants to inform their refinement is important. Within implementation science, evidence syntheses are beginning to answer the question *what is missing* (16–18), proposing key concepts, such as the health systems' architecture, previously overlooked in frameworks (16). These questions can advance existing theory as well. For instance, studies have provided evidence that additional constructs may improve the explanatory power of the Theory of Planned Behavior (19, 20). They suggest that constructs such as self-identity and past behavior may improve the prediction of behavior (21). By asking these questions, everyone can contribute to advancing existing theoretical products.

*How* refers to explanations of causes, consequences, mechanisms, and conditions. Theoretical products describing *what* have outpaced explanations of *how* in implementation science, as evidenced by numerous determinant frameworks but fewer explanatory theories (22). However, empirical enquiries often attempt to establish causes and consequences and, more recently, mechanisms (23–25). Evidence syntheses can assess the evidence for postulates in existing middle-range theories or propose novel theoretical explanations based on evidence. For instance, Meza and colleagues synthesized evidence for the relationship between first-level leadership and inner-context and implementation outcomes (26). They found support for some postulates in existing leadership theory, such as the positive influence of first-level leadership on organizational and implementation climate (27). But also identified limited and inconsistent evidence supporting the commonly regarded postulate that first-level leadership influences implementation outcomes. Individual studies can also contribute to explanations of *how* by directly testing the postulates of theory to evaluate their validity. For instance, Williams and colleagues designed a study to test several postulates of the theory of strategic implementation leadership and articulated how their findings would support or challenge the validity of those postulates (24).

Individual studies can also develop novel explanations of *how* using situation-specific micro-theories. Micro-theories can begin with “cheap” theorizing, formulating tentative and narrow postulates to be evaluated and refined by research. Supported postulates can be maintained and their generalizability further tested, while unsupported postulates discarded or refined. Through such a process, micro-theories can inform middle-range theories with greater generalizability.

Developing novel causal explanations can seem daunting. But researchers and stakeholders can contribute to causal explanations. Humans naturally organize events into causes and consequences. Simple tools can support causal thinking. Qualitative interviewing can elicit implicit causal explanations and coding can characterize those relationships. Linguistic expressions, such as *because* and *since,* shed light on causal conceptualizations (28). The word *because* helps to differentiate a central concept from its determinants. “*I knew that administering the screener (central concept) was a priority because its administration was being measured (determinant*).” Stakeholders can participate in reasoning exercises to clarify their causal thinking. For instance, through counterfactual reasoning, stakeholders can imagine what could have or what may have happened during implementation. This provides answers to questions such as, “*how would implementation have been different if there was consumer demand for the innovation?”* Drawing on direct experience or observations, if-then statements can organize causal thinking. “*If a mandate is instated (cause), then the screening will be administered (effect), but only if screening materials are available (necessary condition).”* We illustrate the application of these tools to prioritize determinants.

*Who, where, and when* refer to boundaries of a theory's generalizability. Theoretical products are developed within limited contexts and their generalizability is tested when applied outside of that context (29). Empirical research can inform how broadly theories should be applied. Boundary conditions, such as conditions of time and space (30), describe the limits of the generalizability of theoretical assumptions (9). Theorizing about boundary conditions can move us beyond selecting familiar theories, to those best suited to a context. Testing moderators of theoretical postulates can also inform boundary conditions. For instance, implementation theories suggest that implementation climate is a driver of innovation use (31–33). Williams and colleagues found the relationship between implementation climate and evidence-based practice use was contingent on a positive molar climate, suggesting that positive molar climates may be a boundary condition under which implementation climate has the strongest effects (34). Applying theory in research outside of the original context in which it was developed can also elucidate boundary conditions. For instance, research can speak to whether the postulates of COM-B (Capability, Opportunity, Motivation and Behavior) (35) hold true across diverse populations, types of behaviors, and in novel contexts. In instances where these postulates are not supported, researchers are encouraged to speculate about potential theoretical boundaries to advance the precision by which we select and apply theory.

## 3. Theorizing about implementation determinants

Efforts to identify implementation determinants frequently surface dozens (36), producing a formidable task of deciding which to target. Existing methods, such as prioritizing determinants deemed important and feasible to address (37, 38), treat determinants as independent, ignoring their complex relationships that may inform their importance. An overly simplistic understanding of how intervention characteristics, implementer activities, and the contextual conditions jointly influence implementation limits our understanding of the key (clusters of) determinants to prioritize. Developing a micro-theory of how determinants unfold can help to organize these complex relationships.

**Figure 1:**
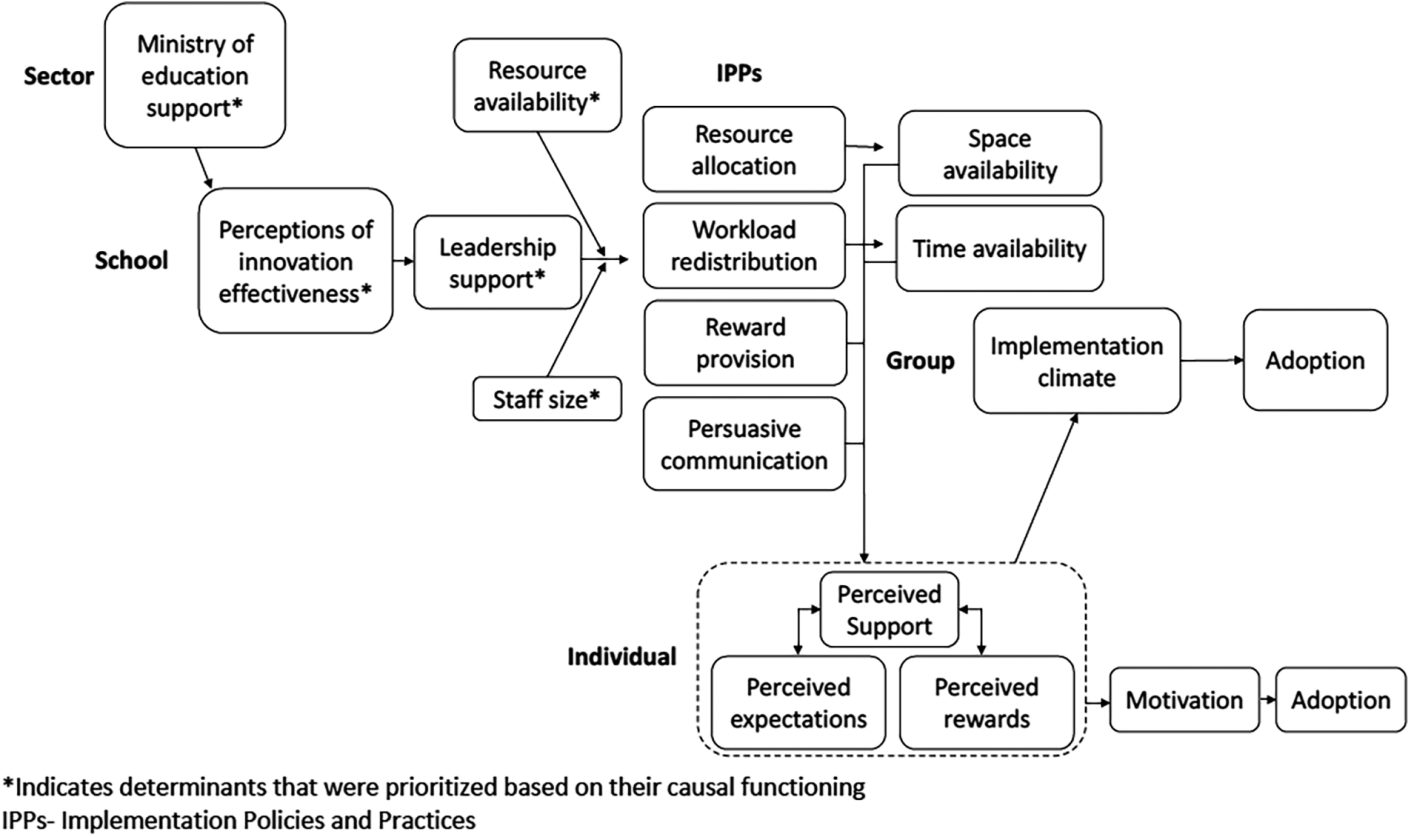
A micro-theory of implementation determinants.

Figure 1 provides an illustration of a micro-theory of how determinants influence school and teacher adoption of a group-based intervention, informed by the questions what, how, who, where and when. We illustrate our approach to stimulate theorizing, not to suggest it be followed as a recipe. We drew on qualitative interviews with stakeholders (teachers and principals) from schools following a phase of implementation-as-usual. Originally, qualitative interviews were used to identify all determinants, stakeholders prioritized determinants based on feasibility and importance, and strategies were aligned with those determinants. Here, we reapproach that process to prioritize determinants based on their causal functioning.

### 3.1. What determinants and IPPs influenced adoption?

Using qualitative interviews, we identified the presence of determinants and implementation policies and practices (IPPs) that schools used to support adoption (39). We inductively coded concepts that emerged, and when aligned, used a combination of determinant frameworks and the theory of organizational determinants of effective implementation (39) to provide a common terminology and conceptual clarity to emergent concepts.

### 3.2. How did determinants and IPPs unfold to influence adoption?

We examined transcripts for linguistic expressions that described the nature of relationships between concepts. If using this approach *a priori*, interviews could be designed to ask about causal explanations. In our post-hoc approach, we looked for terms like *since* and *because* to indicate causal explanations (e.g., I had time to attend the training because our principal asked the deputy teacher to cover my class)*.* This produced many antecedent–outcome linkages (e.g., workload adjustment provided teachers with time for intervention activities) and pointed to moderators (e.g., supportive leaders allocated space for delivery, but only when classrooms were available).

Qualitative responses will undoubtedly lack precision in the chain-of-events that occur between antecedents and outcomes. For instance, rewards were described as influential for adoption, without explanation. To expand on these, the research team constructed if-then statements based on impressions from observations (e.g., if a counselor was recognized by their principal (reward), then this would enhance their motivation (motivation), and increase their likelihood of delivering the intervention (adoption). We used counterfactual reasoning to theorize about the effect of events that did not happen to identify necessary conditions [e.g., if the ministry of education had not signaled support (*necessary condition*), leaders in each school would not have engaged].

We drew on existing theoretical postulates to inform the integration of antecedent–outcome linkages. For instance, interviews suggested several linkages between different IPPs and the perception that implementation was expected, supported, and rewarded (i.e., implementation climate). Drawing on theory (31, 39), we conceptualized IPPs as having an additive effect (i.e., the more IPPs present, the stronger the influence on adoption) and a compensatory effect (i.e., the presence of some high quality IPPs can compensate for the absence or low-quality use of others).

A participatory approach could be used throughout these steps. For instance, initial antecedent-outcome linkages could be presented to stakeholders for member-checking and stakeholders could co-develop if-then statements and engage in counterfactual reasoning [e.g., if X had (not) happened, what do you think would result?] to expand on gaps in the causal chain-of-events.

### 3.3. For whom, where, and when do postulates apply?

A primary function of considering boundary conditions of a situation-specific micro-theory is ensuring its applicability across the contexts it is applied. Including extreme cases is one way to do this. We purposively sampled from schools with varied characteristics (e.g., small and large staff sizes) to surface explanations across diverse characteristics. We modified explanations to be valid in schools with the most extreme characteristics. For instance, we added staff size as a moderator because leadership support only led to workload redistribution in schools with a moderate-to-large staff size*.* A micro-theory will inherently be bounded within a narrower context. As their postulates are empirically supported in new contexts or refined, they can inform middle-range theories.

### 3.4. Prioritizing determinants based on functioning

Determinants can be prioritized based on their theorized influence (see Figure 1). For instance, we may prioritize those occurring early in the causal chain of events that have a cascading influence (e.g., perceptions of innovation effectiveness), moderators that could diminish the effects of other targeted determinants (e.g., resource availability), or necessary determinants that would preclude successful implementation (e.g., Ministry of Education support).

## 4. Using multiple working hypotheses to support theoretically informative research

The tools discussed so far can be used to leverage empirical research to develop novel theory or refine existing theory. An equally important part of theorizing is validating existing theory. Validating theory should push us toward strong theory, while its invalidation should push us to move away from or refine existing theory. With over one hundred theoretical products available (40), their utility must be tested to lead the field toward high value theories. While a couple of theoretical products are most commonly used, the criteria for selecting them is inconsistent (15). The lack of information on the value of theories may maintain the use of familiar theories “without thought or reflection.” (4) We argue, as has been argued for decades before us, that leveraging multiple working hypotheses can produce research that guides the field toward high value theories (11, 41).

Hypotheses are driven by the postulates of theory, whether that theory is explicit or implicit. Platt argued the most rapid scientific advances can be made using multiple hypothesis generation followed by careful experimental design that arbitrates between hypotheses (41). With a single hypothesis, we can only affirm and refine a single theory that may or may not be a reasonable approximation of reality. Imagine the scientific process as a tree diagram with a single path to follow. We might be able to meander down various smaller paths, but we leave other branches unexplored. If, instead, we introduce multiple plausible hypotheses we open all branches we can generate. Good experiments will produce findings consistent with some families of hypotheses, but more importantly, they provide results inconsistent with others. An iterative process of this kind is more efficient in pushing the field toward theories with greater explanatory power and protect against uncritical and superficial theory application. The existence of multiple competing hypotheses in the literature is a sign of health for the field.

Modern statistical analyses have provided tools to evaluate the plausibility of multiple competing hypotheses or models through approaches such as structural equation model fit comparisons and Bayesian alternatives to null hypothesis significance testing. For instance, Bayesian statistical approaches can be used to estimate the posterior probabilities of several competing models given the data, and models with the greatest probabilities can then be selected as the starting point for additional model development. Unfortunately, even moderately complex models may require large sample sizes (*N* > 500) to correctly reach a true model among competing options (42).

As the field responds to growing calls for mechanism-based explanations (43, 44), this will be an important place to adopt multiple hypotheses. Among behavior change theories, there are numerous postulated pathways through which behavior change occurs. For instance, COM-B proposes that capability, opportunity and motivation produce behavior, which in turn influences these components (35). In contrast, the Theory of Planned Behavior posits that attitude toward a behavior, subjective norms, and perceived behavioral control, together shape an individual's behavioral intentions, which, in turn, shapes behavior (19). Rather than proposing hypotheses intended to test the postulates of a single theory, we can compare the explanatory power of each theory in a single study. This approach can also be used to pit multiple novel competing theoretical explanations that emerge through theorizing against one another. This allows for “cheap” theorizing in which we produce many explanations and allow evidence to push us towards those of value. Above all, theorists should feel empowered to readily eliminate unsupported hypothesized determinants or poorly fitting theories.

## 5. Discussion

Complexity is the rule, not the exception, in the change efforts we undertake in implementation science. The classification and organization of constructs into frameworks, delineation of concepts, and theories that explain and predict implementation processes have contributed to creating order and clarity within this complexity (1). Many have argued for the relevance of theory to even the most practical among us who undertake change efforts (45). We agree and also argue that everyone can play a part in advancing theory. All research is related to theory and relevant for pushing theory forward. While many implementation scientists may not identify as philosophers of implementation science, we all play a direct role in theory advancement.

We offer three recommendations for increasing engagement in theorizing. First, articulate the contribution a study can make to advancing or modifying existing theory. To do so, studies must be designed to *question* the postulates of existing theory (i.e., what, how, who, where, when) and findings interpreted in terms of their support for, *or against,* those postulates. Second, engage in novel, “cheap”, micro-theory development. The field is increasingly moving towards articulating causal pathways to open the “black box” of implementation (14, 46, 47). This will require greater engagement in developing theories of the problem (e.g., how determinants unfold to influence implementation) and of the solution (e.g., how strategies can address determinants). We advocate for “cheap” theorizing, in which researchers are empowered to draw on empirical evidence to formulate tentative and narrow postulates to be evaluated and refined by research. As these micro-theories are tested and refined through empirical enquiry, they can inform the foundation of generalizable middle-range theory. Third, to continue advancing existing and novel theories forward, researchers should adopt multiple working hypotheses that pit competing explanations against one another. This approach ensures that our theories do not stay stagnant in their nascent and tentative forms and pushes the field towards high value theories.

One barrier to theorizing that we do not address is funder's expectation for studies to adopt existing theory. The popularity of particular theories, despite a lack of strong empirical support has long been an issue (11). Therefore, we urge that theory not be judged on its longevity or popularity, but on its empirical foundation. If theories developed “in-house” have a strong empirical basis and are being advanced by additional empirical enquiry, this is a benefit to the field. Theory development is never done.

We have sought to clarify how theorizing is for everybody and to demonstrate how the questions we ask and hypotheses we test contribute to theoretically informative research that advances theory. Drawing more people into the process of theorizing is precisely how we push our field towards the advancement and elevation of good theories.

## Data Availability

The original contributions presented in the study are included in the article/Supplementary Material, further inquiries can be directed to the corresponding author.
